# HTLV-1 Specific CD8+ T Cell Function Augmented by Blockade of 2B4/CD48 Interaction in HTLV-1 Infection

**DOI:** 10.1371/journal.pone.0087631

**Published:** 2014-02-05

**Authors:** Chibueze Chioma Ezinne, Makoto Yoshimitsu, Yohann White, Naomichi Arima

**Affiliations:** 1 Division of Hematology and Immunology, Center for Chronic Viral Diseases, Kagoshima University Graduate School of Medical and Dental Sciences, Kagoshima, Japan; 2 Department of Hematology and Immunology, Kagoshima University Hospital, Kagoshima, Japan; 3 Department of Medicine, University of the West Indies, Mona, Kingston, Jamaica; Karolinska Institutet, Sweden

## Abstract

CD8+ T cell response is important in the response to viral infections; this response though is regulated by inhibitory receptors. Expression of inhibitory receptors has been positively correlated with CD8+ T cell exhaustion; the consequent effect of simultaneous blockade of these inhibitory receptors on CD8+ T cell response in viral infections have been studied, however, the role of individual blockade of receptor-ligand pair is unclear. 2B4/CD48 interaction is involved in CD8+T cell regulation, its signal transducer SAP (signaling lymphocyte activation molecule (SLAM)-associated protein) is required for stimulatory function of 2B4/CD244 on lymphocytes hence, we analyzed 2B4/CD244 (natural killer cell receptor) and SAP (signaling lymphocyte activation molecule(SLAM)-associated protein) on total CD8+ and HTLV-1 specific CD8+T cells in HTLV-1 infection and the effect of blockade of interaction with ligand CD48 on HTLV-1 specific CD8+ T cell function. We observed a high expression of 2B4/CD244 on CD8+ T cells relative to uninfected and further upregulation on HTLV-1 specific CD8+ T cells. 2B4+ CD8+ T cells exhibited more of an effector and terminally differentiated memory phenotype. Blockade of 2B4/CD48 interaction resulted in improvement in function via perforin expression and degranulation as measured by CD107a surface mobilization on HTLV-1 specific CD8+ T cells. In the light of these findings, we thus propose an inhibitory role for 2B4/CD48 interaction on CD8+T cell function.

## Introduction

The Human T-lymphotropic virus type 1 (HTLV-1) is implicated in the highly aggressive malignancy, adult T-cell leukemia/lymphoma (ATLL). HTLV-1 infection has a worldwide distribution with endemic areas in Japan, Africa, Caribbean, Central and South America, where majority of infected individuals remain asymptomatic carriers (ACs) and a minority develop a hematologic or neurologic manifestation, ATLL or HTLV-1 associated myelopathy/tropical spastic paraparesis (HAM/TSP) respectively[1]–[6].

In viral infections, however, CD8+ cytotoxic T lymphocyte (CTL) function is central to immune response, mediating effective clearance of infected and transformed (pre-malignant) cells; virus-specific CD8+ T cells also play a role in immune surveillance in HTLV-1 leukemogenesis [7]. CTL dysfunction, however, results in viral persistence [8]–[10]. Constant antigenic stimulation due to chronic hyper-antigenemia in the context of viral persistence induces T-cell exhaustion, a state characterized by impaired CTL function [11]–[14]. This can be attributed in part to the presence of co-inhibitory markers involved in modulating T-cell response to infection [15], [16]. In mouse models of chronic viral infection with lymphocytic choriomeningitic virus (LCMV) infection, CTLs demonstrated increased expression of co-inhibitory receptors and reduced cytolytic function as has been reported for Hepatitis B virus (HBV), Hepatitis C virus (HCV) and Human immunodeficiency virus infections (HIV-1) in humans. The interaction of these receptors with their ligands results in reduced T cell function and ligand blockade improved CTL function in the different viral infections [11], [14], [16]–[19].

2B4/CD244, a member of the signaling lymphocyte activation molecule (SLAM) family of CD2 related receptors is upregulated in chronic viral infections [12], [16], [20], [21]. 2B4 is the only SLAM family receptor known to have variable interactions with its known ligand CD48. 2B4 is expressed on natural killer (NK) cells, CD8+ T cells, basophils, monocytes and eosinophils [22]. The ligand, CD48, is a glycophosphatidyl anchored receptor with high affinity for 2B4 expressed on both lymphoid and myeloid cells and known to be involved in modulation of CTL function. CD48 is upregulated on B-cells in Epstein-Barr virus (EBV) infection and down regulated in HIV infected cells [23]–[25]. Ligation of the 2B4 receptor by CD48 has been shown to be involved in the development of lytic activity on T cells, however, it is not always clear whether ligation results in inhibitory or stimulatory effect on CTL activity due to conflicting findings from existing studies and the discovery of SAP (SLAM-associated protein), a post receptor intracellular adapter expressed on natural killer (NK) cells, T-cells and involved in signal transduction of SLAM family members, including 2B4 and CD48.

2B4-CD48 interaction has been variably shown to either activate or inhibit effector function; this however depends on levels of SAP expression; in the presence of insufficient SAP or its absence, inhibitory and stimulatory if high. Increased 2B4 receptor expression or CD48 ligand density could also render SAP limiting [26]. The interaction of these receptors with their ligands results in reduced T cell function and blockade of this interaction improved CTL function in the different viral infections [11], [14], [16]–[19].

Existing studies tend to focus on 2B4 expression on NK cells with less emphasis on the role of 2B4 on CTL function. Previous results from our laboratory have demonstrated an impaired CTL response in HTLV-1 infection resulting in viral persistence, partly due to increased expression of the PD1 receptor (programmed cell death-1); blockade of interaction with its ligand PDL-1 resulted in improved effector function [27]. Studies on blockade of PD1/PDL1 interaction have been extended to clinical trials and validated as an important target for therapeutic intervention due to tumor regression and disease stabilization in advanced cancer patients [28], thus, it is of interest to know the role of 2B4-CD48 interaction on CTL function as such knowledge could inform novel therapeutic strategies or clearance of HTLV-1 infection, prevention of leukemic transformation in ACs and treatment of ATLL.

In the present study, we compare 2B4 expression among healthy donors (HDs), clinically asymptomatic carriers (ACs), patients with ATLL and describe its role as a co-inhibitory receptor and marker of CTL exhaustion in ATLL and AC via measurement of CD107a degranulation activity and perforin expression.

## Materials and Methods

### Ethics Statement

The medical Ethics Committee of Kagoshima University, Kagoshima, Japan, approved this study involving human participants. All subjects gave written informed consent. All investigations were in line with the principles contained in the Declaration of Helsinki.

### Study Subjects

Blood samples from 52 HTLV-1 seropositive patients (21 adult T-cell leukemia/lymphoma (ATLL), 31 asymptomatic carriers (AC)) and 12 HTLV-1 individuals, all residents of Kagoshima, Southern Kyushu were collected (Table 1). They all gave written informed consent to participate in this study. The study protocol was reviewed and approved by the medical ethical committee of Kagoshima University; characteristics of the study group are outlined in Table 1. PBMCs (peripheral blood mononuclear cells) were isolated by Ficoll Hyphaque centrifugation (Lonza Walkersville, Walkersville, MD) from heparinized whole blood samples and preserved in liquid nitrogen until use. All patients selected were either HLA-A*0201 positive or A*2402 positive as these are the major immuno-dominant epitopes in HTLV-1 infection in our study population [29]. The number of samples with detectable Tax-specific CD8+ T cells in all samples used in this study is summarized in Table 2.

**Table 1:** Characteristics of the Study Groups.

Disease status	Number	Age(mean)	Sex(M/F)^a^
ATLL^b^	21	40 s–70 s	10/11
AC^c^	31	50 s–70 s	8/23
HD^d^	12	30 s–60 s	3/9

a M/F = male/female,

b ATLL = adult T-cell leukemia/lymphoma,

c AC = asymptomatic carrier.

d HD = healthy donor.

**Table 2:** Detectable Tax-specific CD8+ T cells in all samples used in this study.

Tax/HLA^a^ tetramers used in this study			
	Disease status		
	AC	ATLL	HD
HLA-A0201/Tax 11–19	10/31	7/21	
HLA-A2402/Tax 301–309	18/31	13/21	
No. of tetramer^+^ samples/total no. of blood samples	28/23(90.3%)	20/21(95.2%)	
HLA-A*0201/CMV^b^ pp65	6/31	4/21	3/12
HLA-A*2402/CMV tetramer	16/31	8/21	5/12
No. of tetramer^+^ samples/total no. of blood samples	22/31(71%)	12/21(57.1%)	8/12(66.7%)
HLA-A*0201/EBV^c^ tetramer BMLF-1	6/31	4/21	1/12
HLA-A*2402/EBV tetramer BRLF-1	10/31	6/21	4/12
No. of tetramer^+^ samples/total no. of blood samples	16/31(51.6%)	10/21(47.6%)	5/12(41.7%)

a human leucocyte antigen.

b cytomegalovirus.

c epstein-barr virus.

### Cell Surface Staining

Cryopreserved PBMCs were thawed, washed with staining buffer (containing 10% Fetal cows serum and 0.1% sodium azide (NAN_3_)) and then stained for antibodies to the following: CD8, CD4 (BD, San Diego, CA), 2B4 (clone C1.7, Biolegend (San Diego, CA), CD48 (Clone MEM-102 Exbio Praha, Vestec CZ) and appropriate isotypes incorporated as controls. Surface 2B4 expression was then estimated by subtracting the percentage of isotype control positive cells from the percentage of anti 2B4 positive cells [30]. Acquisition was done using FACS Scan (BD Biosciences, Mountain View, CA, USA) or FACS Calibur (BD Bioscience, San Jose, CA, USA) flow cytometer and analyzed with FlowJo Treestar software (Tree Star, San Carlos, CA, USA). For analysis of 2B4 expression on virus- specific CD8+ T cells by flow cytometry, the following HLA- A restricted tetramers were used: all phycoerythrin (PE) -conjugated HLA-A*0201/Tax 11–19 LLFGYPVYV, HLA-A*2402/Tax 301–309 SFHSLHLF, HLA-A*0201/CMV (NLVPMVATV), HLA-A*2402/CMVpp65 (QYDPVAALF), HLA-A*0201/EBV BMLF-1(GLCTLVAML), HLA-A*2402/EBV BRLF-1(TYPVLEEMF) (All MBL (Medical and Biological Laboratories) Nagoya, Japan). To evaluate maturational phenotype of 2B4 expressing CD8+ T cells, PBMCs were stained with antibodies to the following: CD8 (BD, San Jose, CA), 2B4 (clone C1.7), CD57, CD62L (all Biolegend, San Diego, CA), CD45RA, CCR7 (both eBioscience) antibodies, acquired and analyzed by flow cytometer (FACS Calibur). Matched isotypes were incorporated and used as controls in all experiments.

### CD 107a Degranulation Assay

Analysis of CD107a cell surface mobilization to determine cell-mediated cytotoxicity of CD8+ T cells was quantified using the flow cytometer. PBMCs from ATLL and AC subjects known to display considerable HTLV-1 specific clusters to HLA-A*0201/Tax 11–19 and HLA-A*2402/Tax 301–309 were suspended in medium, RPMI 1640 (supplemented with 10% heat-inactivated FCS, 100 Uml penicillin, 0.1 mg streptomycin) and cultured in a 96 well plate in a 5% CO2 incubator at 37°C for 4 hours with or without 0.02 µM HTLV-1 Tax peptide (Sigma-Aldrich, Japan) in combination with CD107a/IgG1 isotype MAbs-FITC, secretion inhibitor monensin (Immunocyto CD107a detection kit, MBL, Japan). Anti-CD48 antibodies (5 ug/ml) were also added to the culture. Harvested cells were washed and further stained with HLA- tetramer CD8PerCP (BD Bioscience, San Jose, CA). About 1×10^5^ CD8+T cells were acquired by FACScan or FACS Calibur flow cytometer and analyzed with Flowjo Software.

### Intracellular Perforin Staining

PBMCs were cultured in 96 well plate in 5% CO2 incubator at 37°C for 6 hours in RPMI 1640 medium with or without 0.02 µm HTLV-1 tax peptide (Sigma-Aldrich, Japan) and brefeldin A (BD San Diego, CA) at a final concentration of 10 µg/ml. CD48-blocking antibodies were added at a final well concentration of 5 µg/ml [16]. Harvested cells were washed twice with phosphate buffered saline (PBS) and stained with anti-CD8 PerCP (BD Biosciences, San Jose, CA USA) or CD8-PC5 (Cytostat-Coulter, Fullerton USA), PE-conjugated HLA-A*0201 and HLA-A*2402 for 15 minutes at room temperature. Cells were washed and then permeabilized using FACS permeabilizing solution 2 (BD San Diego, CA) for 10 minutes at room temperature. Cells were again washed, suspended in saponin-containing PBS for 10 minutes at room temperature and then incubated with FITC-labeled perforin and isotype control IgG2b κ-FITC (BD) for 20 minutes at room temperature. Finally, cells were washed and analyzed with FACS Calibur flow cytometer and FlowJo Treestar software based on forward and side scatter in the lymphocyte gate as described [31].

### Intracellular SH2D1A Detection by Flow Cytometry

Cryopreserved PBMCS were thawed and washed twice with staining buffer. Surface staining was done with antibodies to HLA-A*0201, HLA-A*2402 tetramers and incubated for 15 minutes at room temperature; further staining was done with antibodies to CD8 and incubated for another 15 minutes. Cells were washed and then permeabilized using FACS Permeabilizing Solution 2 (BD) according to manufacturer’s instructions, washed twice with staining buffer, incubated with unconjugated rat anti-SH2D1A monoclonal antibody ((1D12), Santa Cruz Biotechnology, CA,USA) or rat IgG2a K Isotype control (Biolegend, San Diego, USA) for 20 minutes at room temperature and then stained with FITC- labeled goat anti rat- IgG (Santa Cruz Biotechnology, CA, USA) for 20 mins at room temperature as described [18], [32]. Cells were washed and re-suspended in 1% paraformaldehyde and kept on ice until analysis by flow cytometry (FACS Calibur, BD Biosciences, San Jose, CA). 1×10^5^ CD8+ T cells were acquired.

### Statistical Analysis

Differences were analyzed by Mann-Whitney U test and Wilcoxon matched pairs test using GraphPad Prism 6 (v6.01c). p-values less than 0.05 were considered significant.

## Results

### 2B4 Expression on Total CD8+ Lymphocytes

**Figure 1:**
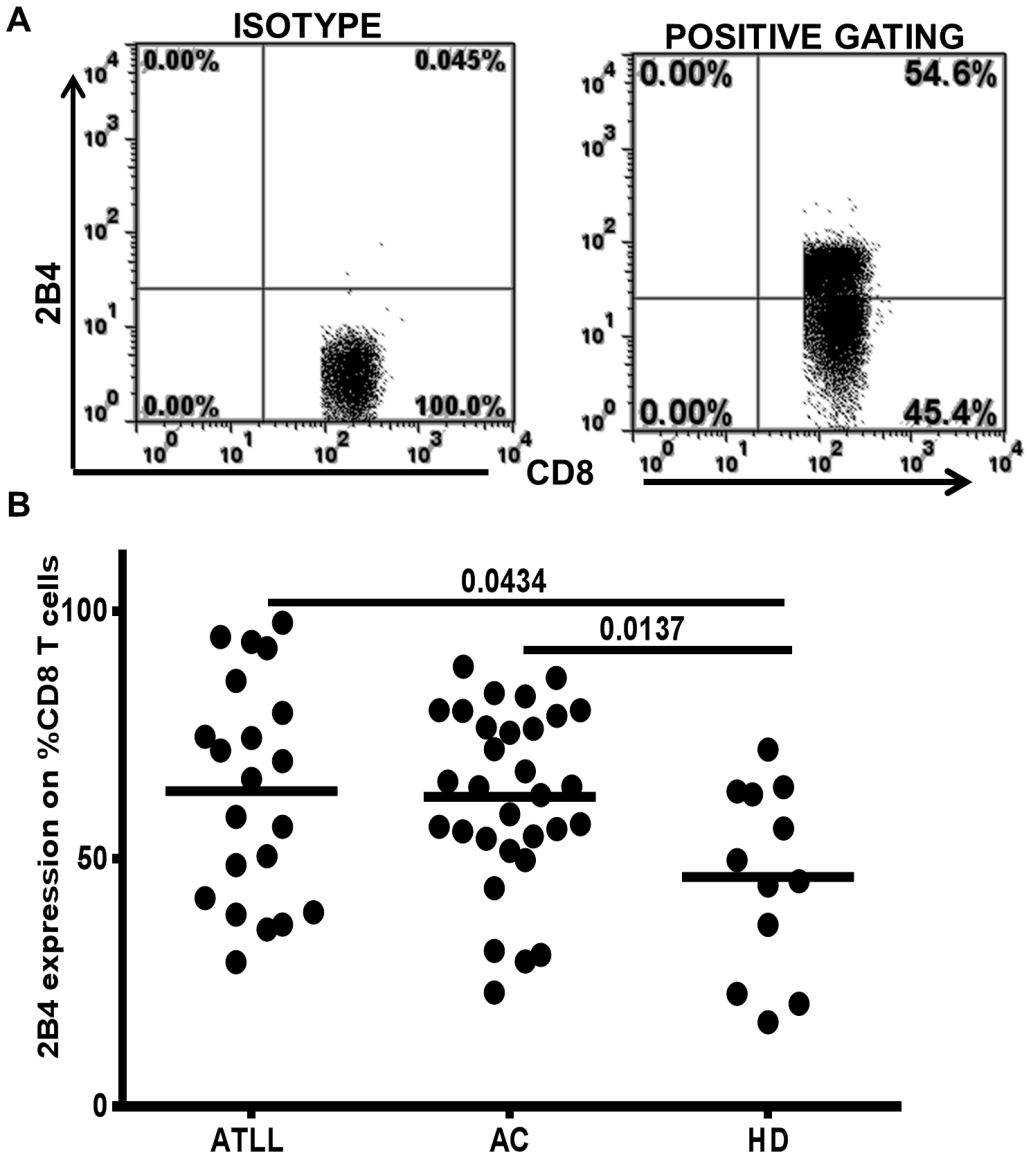
2B4 expression on total CD8+Tcells in HTLV-1 infection. Appropriate isotype controls were used to set quadrant gates, A) Dot plot of isotype and positive 2B4 staining in a representative sample. Numbers indicate frequency of 2B4 expression on total CD8+ T cells of an HTLV-1 infected subject B),Summary data showing 2B4+ expression on total CD8+ lymphocytes in 52 HTLV-1 infected subjects (21 ATL and 31 AC) and 12 uninfected healthy donors (HD). Each dot represents a single individual. Horizontal bars represent mean values (p-value based on Mann-Whitney test).

To determine the expression of 2B4 on total CD8+ T cells, we analyzed PBMCs (peripheral mononuclear blood cells) from HTLV-1 infected patients (ATLL (adult T cell lymphoma/leukemia) and ACs (asymptomatic carriers)) relative to samples from uninfected individuals. 2B4 was detected on total CD8+ T cells and significantly higher in HTLV-1 infected compared to uninfected. Figure 1 A, B shows a representative dot plot and summary data of all individuals (frequency of 2B4 expression on total CD8+ T cells; ATLL: 63.7±22.2; AC: 62.6±17.9; HD: 42.6±18.7, mean ± standard deviation (SD)). Significant differences were analyzed in the HTLV-1 infected (ATLL, AC) versus uninfected healthy donors, p<0.05. There was no difference between ATLLs and ACs (ATLL vs. HD, p<0.05; AC vs. HD, p<0.05). No difference was observed between ATLLs and ACs.

### Maturational Phenotype of CD8+2B4+ T Cells

**Figure 2:**
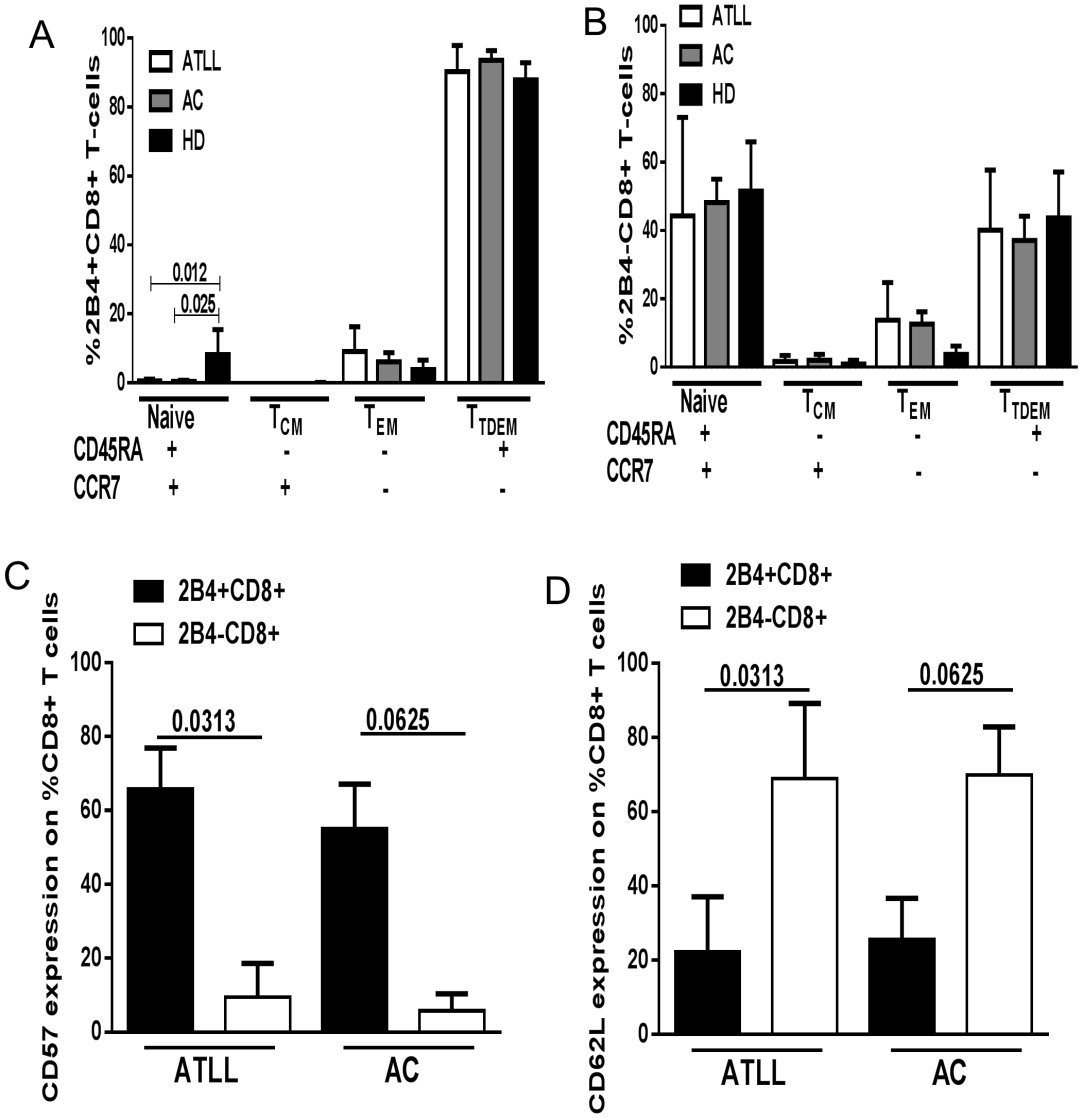
Characterization of memory population of 2B4+CD8+ T cells by CCR7, CD45RA, CD62L and CD57 expression. 2B4+CD8+ T cells tended towards effector and terminally differentiated memory phenotype relative to 2B4-CD8+ T cells. A, B) Summary of frequency of CD45RA and CCR7 coexpression on 2B4+CD8+ and 2B4-CD8+ T cells in HTLV-1 infected (6 ATL and 5 AC) and 5 healthy donors (HD). 2B4+CD8+ T cells expressing CD45RA and CCR7 showed a mature effector phenotype in the effector memory and terminally differentiated compartments compared to 2B4-CD8+ T lymphocytes. C) Summary of frequency of CD57 on CD8+ T cells were significantly higher in 2B4+ compared to 2B4− populations in both ATLL and AC D) Lower CD62L expression on 2B4+CD8+ compared to 2B4-CD8+ T cells. Data for CD57 and CD62L was obtained from 6 ATL and 5 AC. Phenotypic patterns are indicated by legends. Horizontal lines represent mean ± S.D; statistical comparisons, p- value based on Mann-Whitney and Wilcoxon matched-pairs signed rank test.

To determine the pattern of expression of 2B4 on CD8+T cells differentiation in CD8+ T cells from HTLV-1 infected, we examined 2B4 expression relative to CD45RA and CCR7 coexpression. 2B4 was present mainly in the naïve, effector and terminally differentiated memory groups, however, with a preference for terminally differentiated (CD45RA+CCR7− mean 87% ±9.9%) and effector memory (CD45RA-CCR7-mean 7.8% ±5.4) compartments. In the naïve compartment, 2B4+CD8+T cells were significantly higher in uninfected healthy donors (HD) compared to ATLL and AC (ATLL 0.59±0.53; AC 0.38±0.38; HD 8.22±7.2; p<0.05) possibly suggesting ongoing activation in response to exposure to viral infection. Comparing terminally differentiated cells (CD45RA+CCR7−) in 2B4+ CD8+ T cell population to 2B4-CD8+ T-cell population revealed a higher frequency in 2B4+CD8+ T cells population (p<0.05); in contrast a higher proportion of naïve cells (CD45RA+CCR7+) was noted in 2B4− CD8+ T cells relative to 2B4+ CD8+ T cells (P<0.05) (Figure 2 A, B).

### Analysis of CD57 and CD62L Expression on 2B4+CD8+ T Cells

2B4+CD8+ T cells also revealed CD57 (61.25% ±12.9%) and CD62L (22.3% ±12.3%) expression. Comparison of 2B4+ and 2B4− CD8+ T cells revealed a higher frequency of CD57 (p<0.05) and lower frequency of CD62L expression (p<0.05) on 2B4+CD8+ T cells in paired samples from ATLL subjects as shown in Figure 2 C, D).

### 2B4 Expression on HTLV-1 Specific T Cells

**Figure 3:**
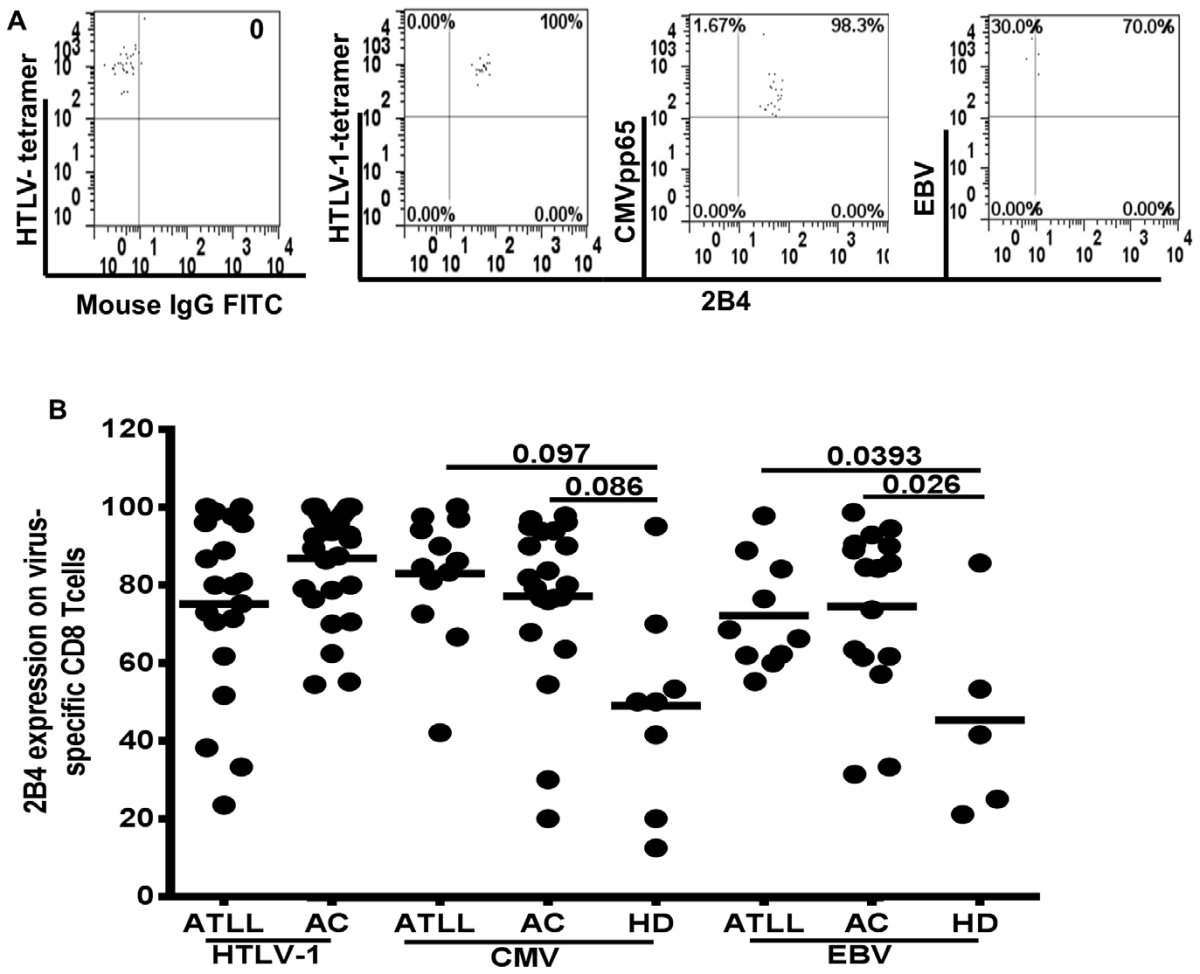
Expression patterns of 2B4 on virus-specific CD8+T lymphocytes. A), Representative dot plots showing gating strategy for 2B4 and percentage of 2B4 expression on virus-specific CD8+ T lymphocytes, HTLV-1, CMV and EBV. B) Summary data of expression of 2B4 on HTLV-1 infected (ATLL, AC); CMV− and EBV-specific CD8+ T cells in ATLL, AC and HD. Horizontal line represents the mean. (p-value based on by Mann-Whitney test).

HLA-A*0201 and *2402 are the more commonly detected epitopes in ACs and ATLLs in the study region [33], [34], thus, we evaluated the frequency of 2B4 expression on HTLV-1-specific CD8+ T cells corresponding to the two major immuno-dominant epitopes, HLA-A*0201 (11–19) and HLA-A*2402 (301–309). We detected positivity in both ATLL (75.2±23.1) and AC (86.9±13.7) subjects, however no difference was observed between the two groups neither was any detected in frequencies between the two epitopes in either group (Figure 3).

Similarly, in other latent infections (CMV, EBV) 2B4 was also present on virus-specific CD8+ T cells. 2B4 expression was significantly higher on HTLV-1 infected group compared to virus-specific CD8+ T cells from healthy individuals. No difference in 2B4 expression was detected between ATLL and AC in both CMV− and EBV− specific CD8+ T cells (Figure 3).

### Upregulation of 2B4 Expression on Virus Specific CD8+ T Cells

**Figure 4:**
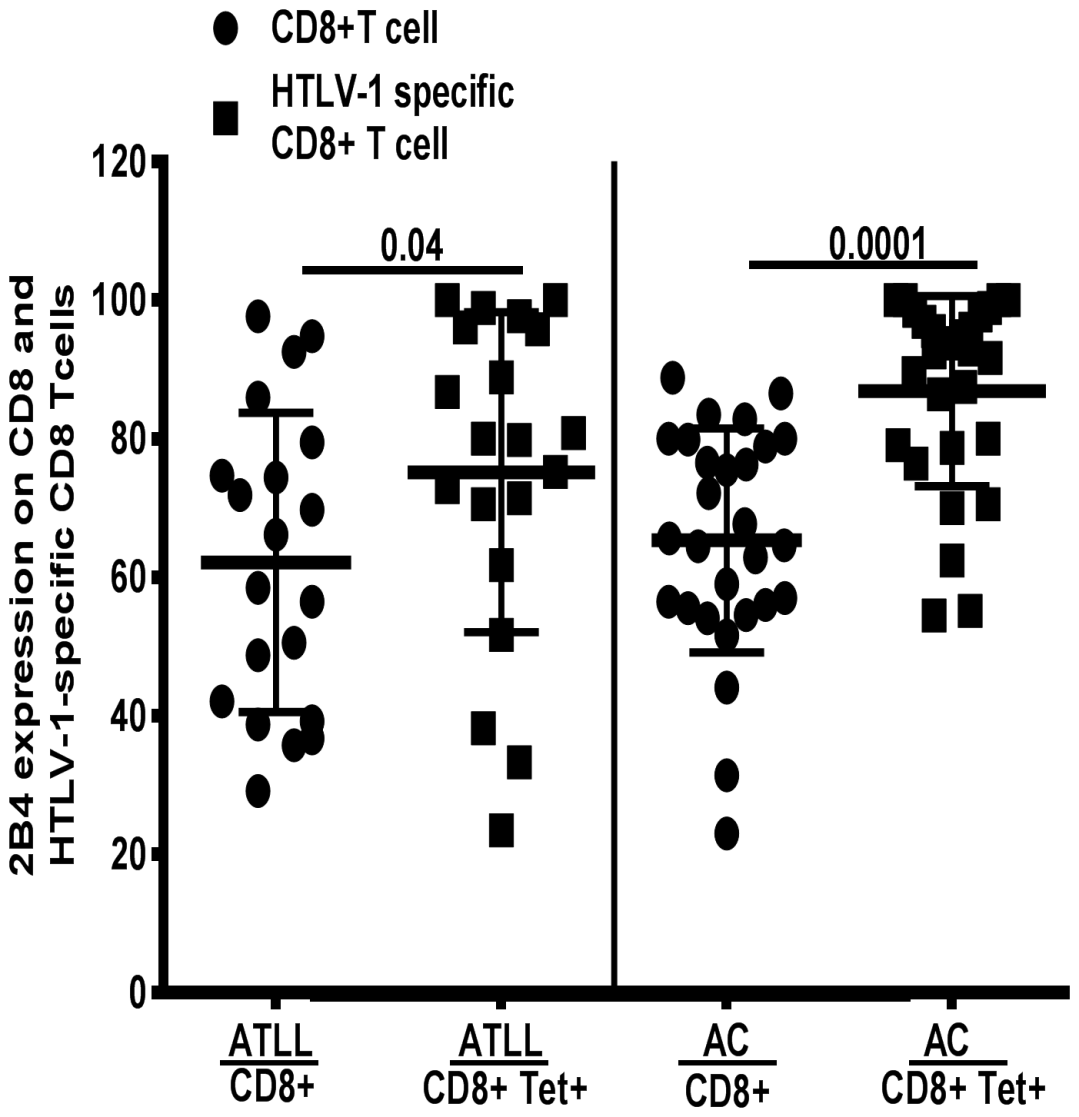
Upregulation of 2B4 expression. Frequency of 2B4 expression on HTLV-1 specific CD8 relative to total CD8+T lymphocytes was analyzed in paired ATLL and AC subjects (20 ATLL and 28 AC). A significant increase in 2B4 expression on virus specific CD8+T cells compared to total CD8+T cells in both ATLL and AC. Mean ± S.D denoted by horizontal lines(p-value based on Wilcoxon matched-pairs signed rank test).

Comparing 2B4 expression on HTLV-1 specific CD8+ T cells relative to total CD8+ T cells in matched ATLL and ACs subjects, we observed a significant up-regulation of 2B4 expression in both groups as shown in Figure 4 (ATLL: 62.2±21.6 vs. 75.2±23.1; AC: 65.3±16.2 vs. 86.9±13.7; p<0.05).

### CD48 Expression on CD4+ T Cells

**Figure 5:**
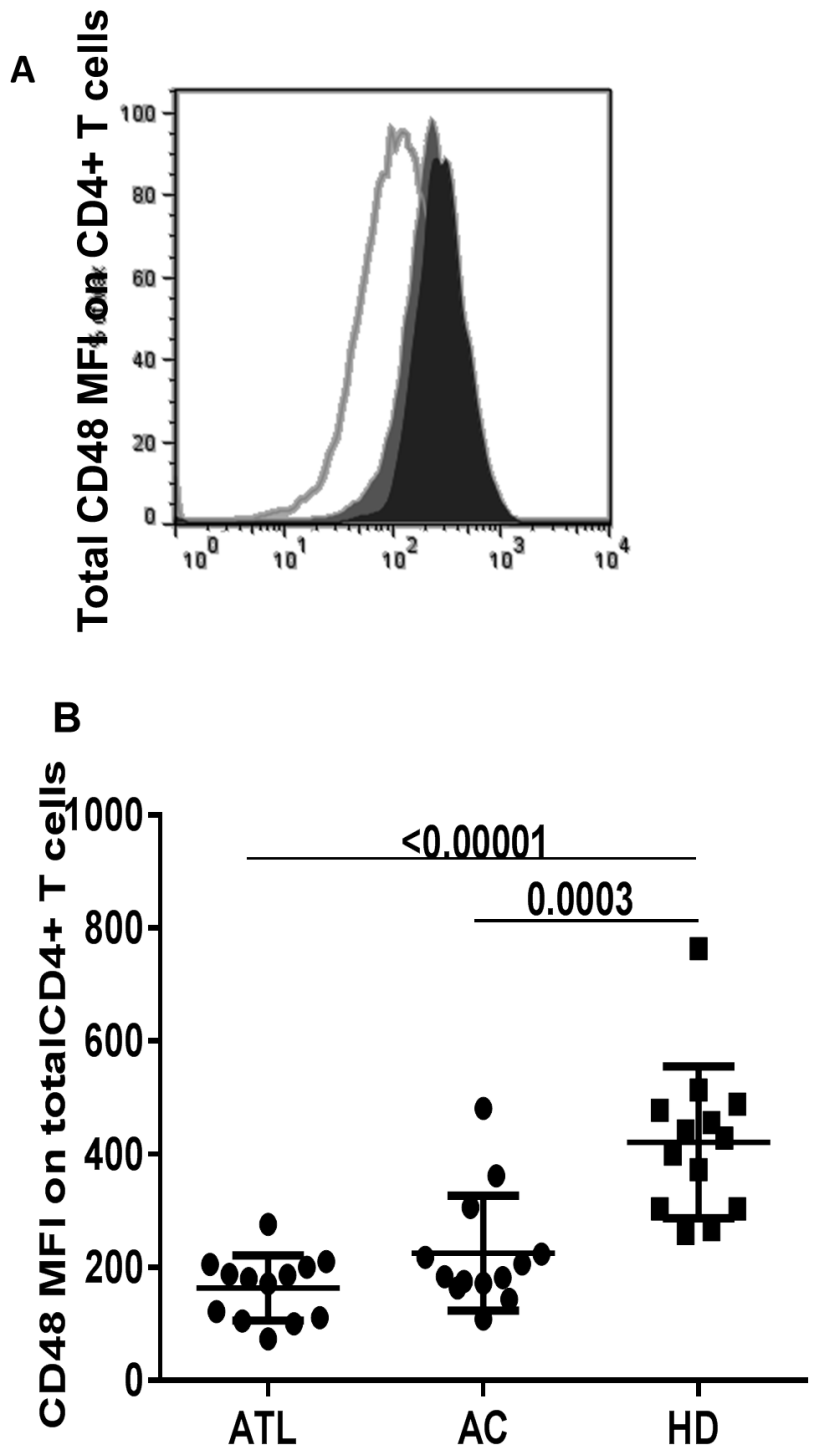
CD48 MFI (mean fluorescence intensity) on CD4 T cells. In A) Representative histograms of CD48 MFI on CD4+ T cell; ATLL (opened histogram), AC (grayed histogram), HD (black histogram) B) summary data comparing CD48 MFI (mean fluorescence intensity) on total CD4+ T cells in HTLV-1 infected and uninfected subjects (13 ATLL, 13 AC and 12HD). CD48 MFI was visibly lower in ATLL and AC relative to HD. The horizontal line represents the mean ± S.D (p-value based on Mann-Whitney test).

CD48, the known 2B4 ligand, a SLAM family receptor is also involved in signal transduction and co-stimulation of immune response. Previous works have shown a variation of CD48 expression with viral infections; downmodulation on HIV-infected and upregulation on EBV infected cells [23], [24], [35]. Thus, we evaluated CD48 expression on CD4+ T cells in HTLV-1 infected and observed a decrease in CD48 MFI compared to uninfected healthy subjects, Figure 5.

### CD107a Degranulation Assay

**Figure 6:**
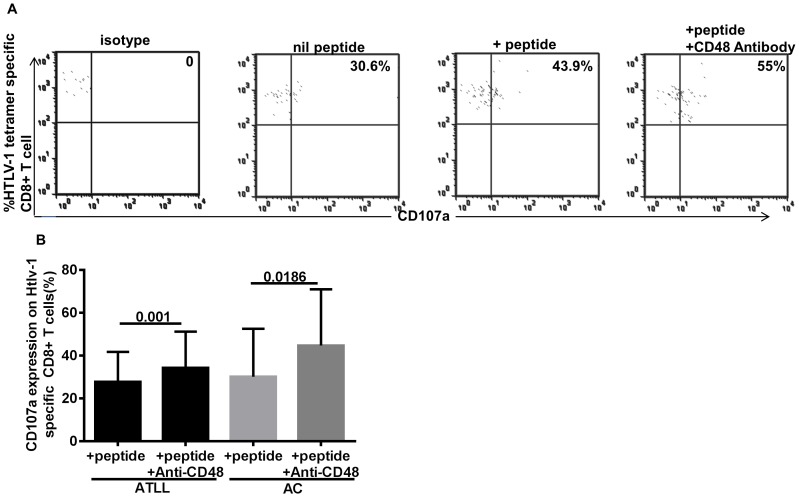
Increased CD107a degranulation of HTLV-1 specific CD8+ T cells on ligand blockade in both ATLL and AC. In A), Representative dot plots show CD107a surface degranulation on HTLV-1 specific CD8+ T cells in varying conditions. In B) Summary plot of CD107a surface degranulation in tax-stimulated samples in the presence and absence of CD48 antibody addition in ATLL (11 independent experiments in 7 patients) and AC (12 independent experiments in 8 subjects). Experiments were done in duplicates (p-value based on Wilcoxon matched-pairs signed rank test).

To further determine the effect of addition of CD48 antibodies on effector function in terms of CD107a degranulation activity, PBMCs from HTLV-1 infected patients were incubated with and without HTLV-1 Tax peptide in the presence of anti CD48 blocking antibodies and the effect on *in-vitro* CTL function measured in HTLV-1 infected (12 independent experiments in 8 asymptomatic carrier (AC) samples and 11 experiments in 7 samples with ATLL). As shown in Figure 6B, we observed a significant increase in CD107a degranulation on HTLV-1 specific CD8+T cells on simultaneous peptide stimulation and anti-CD48 addition (p<0.05).

### Perforin Production on HTLV-1 Specific CD8+ T Cells on Ligand Blockade

**Figure 7:**
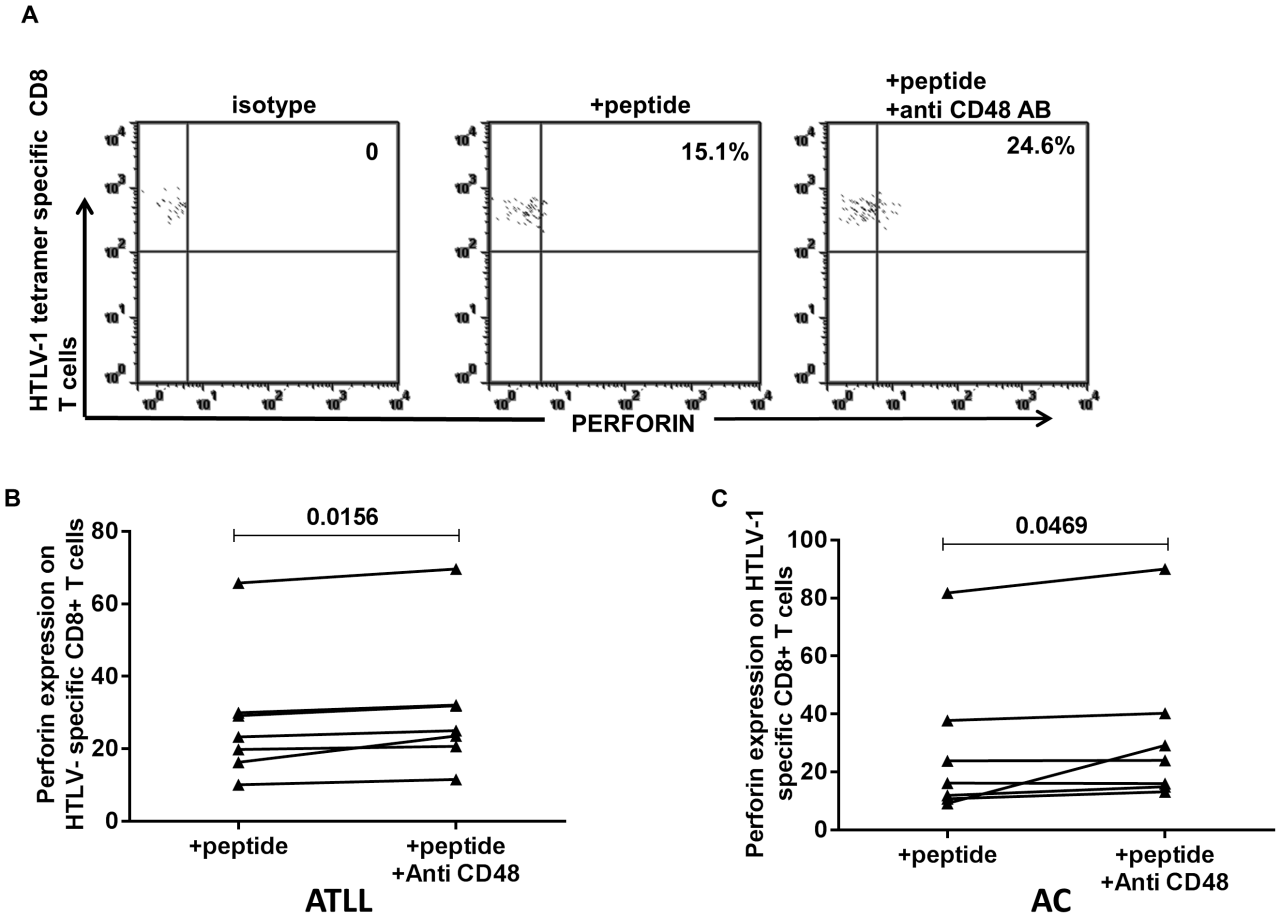
Improved perforin expression on HTLV-1 specific CD8+ T cells on ligand blockade in ATLL and AC subjects. PBMC from 6 ATLL and 5 AC subjects were stimulated for 6 hours with and without HTLV-1 peptide in the presence or absence of CD48 in 7 independent experiments. In a) Representative dot plots showing perforin release in peptide only stimulated and peptide stimulated samples to which anti-CD48 was added. In b) Summary of data for all 6 ATLL subjects in 7 independent experiments. In c) summary of data for all 5 asymptomatic carriers in 7 independent experiments (p-value based on Wilcoxon matched-pairs signed rank test).

Interference in ligand receptor interaction has been shown to modulate effector function; hence, we evaluated the effect of CD48 ligand blockade on effector function via measurement of intracellular perforin production on HTLV-1 specific CD8+T cells. PBMCs from HTLV-1 infected patients were incubated with and without HTLV-1 Tax peptide in the presence of anti CD48 antibodies and the effect on in-vitro CTL response in terms of perforin production measured in 6 ATLL and 5 AC subjects in 7 independent experiments. We observed a weak response with little perforin production on short-term peptide stimulation, however, additional CD48− blockade resulted in enhanced perforin expression (p<0.05) (Figure 7).

### SAP (SH2D1A) on CD8+ T Cells

Levels of SAP expression determine the eventual outcome of 2B4-CD48 interaction in preference to other adaptor proteins. A previous study attributed an activating role to 2B4 in HTLV-1 infection due to high SAP levels, the study demonstrated a significantly higher SAP expression in HAM/TSP individuals compared to asymptomatic carriers although data was only shown for HAM/TSP (a subgroup of HTLV-1 manifestation) and AC subjects.

Data for SAP levels in ATLL is non-existent; hence we quantified SAP expression on CD8+ and virus specific CD8+T cells in HTLV-1 infected (ATLL and AC) to determine the possible contribution of SAP to the inhibitory role observed on HTLV-1 specific CD8+T cells.

**Figure 8:**
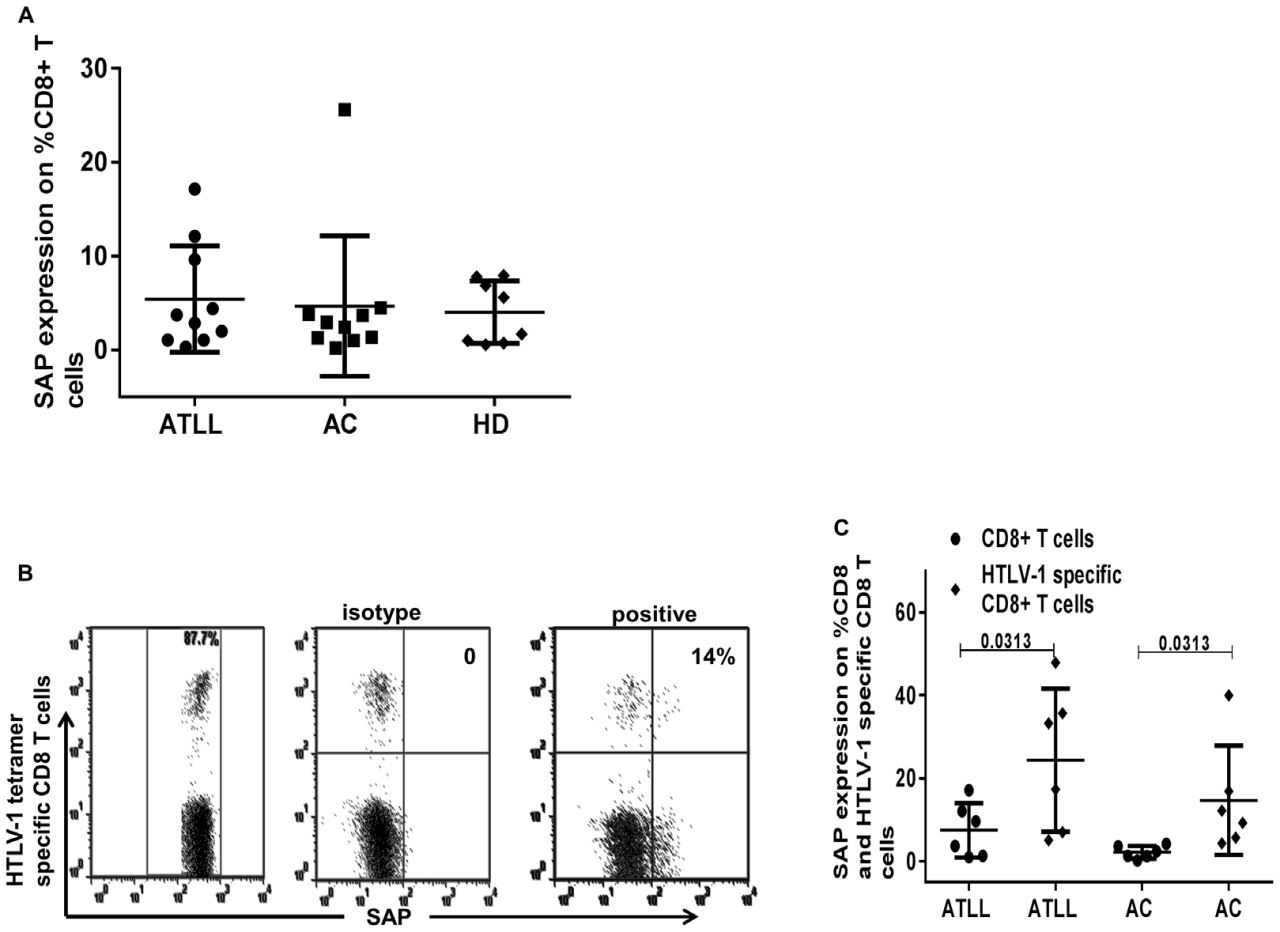
SAP expression on CD8+ T cells and HTLV-1 specific CD8+T cells in HTLV-1 infection. In A) Summary of data for percentage SAP expression on total CD8+ T cells in HTLV-1 infected and healthy individuals. In B) Representative dot plots showing gating strategy for HTLV-1 tetramer positive CD8+T cells, isotype control, and SAP expression on HTLV-1 specific CD8+ T cells. In C) Summary data of SAP expression on subjects for whom HTLV-1 specific cluster could be demonstrated matched with SAP expression on CD8+ T cells in HTLV-1 infected subjects (ATLL (9), AC (8)) (p-values based on Mann-Whitney and Wilcoxon matched-pairs signed rank test).

No difference in SAP expression on CD8+ T cells was observed across all groups, however, levels of SAP on HTLV-1 specific CD8+ T cells were significantly increased in comparison to total CD8+T cells in matched ATLL (9) and AC (8) subjects (Figure 8).

Statistical analyses were performed using Mann-Whitney test for unpaired samples and Wilcoxon matched pairs test for paired samples.

## Discussion

Upregulation of the co-inhibitory marker 2B4 is associated with a decrease in virus-specific CD8+ cytotoxic T lymphocyte (CTL) function in lymphocytic choriomeningitis virus (LCMV) infection in mouse models, HIV, Hepatitis B and Hepatitis C virus infections in humans, chronic HTLV-1 infection is associated with an expansion of CD8+ effector memory T-cells but which are dysfunctional.

We demonstrate here that 2B4 is increased in total CD8+ T cells and further upregulated on HTLV-1 specific CD8+ CTLs in chronic HTLV-1 infection compared to healthy uninfected donors; there is generalized immune dysfunction as indicated by upregulation of 2B4 in CMV− and EBV-specific CTLs in HTLV-1 infected individuals compared to healthy donors, lymphoid homing marker CD62L in 2B4-expressing compared with 2B4-negative CD8+ CTLs in keeping with expansion of the CD8+ terminal effector memory (TEM) T-cell compartment and an increase in activation marker CD57 in 2B4-expressing CD8+ T-cells compared with 2B4-negative CTLs. Altogether, these findings suggest a co-inhibitory function of 2B4, and its upregulation in HTLV-1 infection is associated with CTL dysfunction.

CTLs expressing 2B4 tended to have terminally differentiated and effector memory phenotypes based on flow cytometric analyses of CD45RA and CCR7 expression. These phenotypes have been shown to be associated with impaired CTL function in chronic viral infections, despite upregulation of activation markers in the TEM (terminal effector memory) compartment as shown in our study [36], [37]. The downregulation of CD62L expression observed in these T-cell memory subsets in this study, is known to be associated with decreased proliferative capacity [8], [38]. Furthermore, West et al showed an upregulation of the 2B4 mRNA in exhausted T-cells in the effector memory compartment in chronic viral infection [12], [39]. 2B4+ CD8+ T cells exhibited a high frequency of CD57 relative to 2B4-CD8+T cells and a CD62lo expression, suggesting a relative expansion of CD8+ TEMs.

Also, we observed a higher frequency of 2B4 expression on HTLV-1 tetramer specific CD8+ T cells. Several studies have reported on the immune exhaustion phenotype of HTLV-1 specific CD8+T cells, in keeping with our hypothesis of a possible co-inhibitory function of 2B4 on interaction with its ligand, as well as the expansion of the TEM subset. A similar upregulation of 2B4 expression was observed on CMV− and EBV-specific CD8+ T cells from HTLV-1 infected compared to uninfected individuals.

The observation that most persons with HTLV-1 infection do not progress to disease, more strongly indicates that HTLV-1 infection is mediated by the host immune response to infection, therefore the upregulation of 2B4 in CD8 CTLs may be understood in the context of a homeostatic response to chronic immune activation. This implicates 2B4 in the regulation of CD8+ T cell response in HTLV-1 infection and a possible role in HTLV-1 pathology.

In addition, CTL response in a majority of patients with ATLL is deficient or dysfunctional, this dysfunction is not relegated to patients with ATLL but extends to asymptomatic carriers (AC) and patients with HAM/TSP(HTLV-1 associated myelopathy/tropical spastic paraparesis) [34], [40], [41]. ATLLs have been shown to be in a state of immunosuppression, this has been attributed in part to the expression of PD1,an inhibitory receptor also upregulated on HTLV-1-specific CD8 T cells; blockade of interaction with its ligand PDL1 (programmed cell death-1) resulted in a partial restoration of CTL function [27]. The improvement in CD107a degranulation and perforin expression was also observed on CD8+ T cells in both ATLL and AC. Increased 2B4 expression may limit SAP activity, nonetheless, increased 2B4 receptor expression on HTLV-1 specific CD8+T cells and relatively low SAP levels support the inhibitory role of 2B4/CD48 interaction. This augmentation in effector function on blockade of 2B4/CD48 interaction in both ATLL and AC supports our hypothesis of a possible 2B4-receptor involvement and the probability of the effector cells generated being dysfunctional in part due to increased 2B4 expression. CD8+ T cells from HTLV-1 infected and HTLV-1 specific CD8+T cells have been shown to be exhausted, thus supporting our observation. Similarly, higher levels of 2B4 expression was observed on other persistent infections, CMV− and EBV-specific CD8+T cells from HTLV-1 infected relative to uninfected. Interestingly, CD48 ligand expression was down regulated on CD4+ T cells in contrast to EBV infection. CD48 is implicated in effector function in immune response, varying with infection; increased levels indicating effective immune defense and vice versa [23], [35], [42], thus, probably indicating dysfunction of CD4+T cells in HTLV-1 infection due to persistence of the infection, this could also be a viral attempt at evading immunity.

Our study hereby shows an inhibitory role for 2B4 on CTLs in HTLV-1 infection in both ATLL and AC. However, another study [43] suggested an activating role for 2B4 in HTLV-1 associated myelopathy/tropical spastic paraparesis (HAM/TSP), correlating with SAP levels, which was higher in patients with HAM/TSP compared to asymptomatic HTLV-1 infected individuals, and lowest in uninfected controls. ATLL patients have been shown to display suppressed immune response, in contrast to immune stimulation in HAM/TSP subjects [31], [41], [44]–[46]. Recent studies have shown an increased cytotoxic response to viral invasion in samples from HAM/TSP patients compared to those from ATLL patients with reduced cytotoxic function [47], [48]. Our results showed a similarly high 2B4 expression on HTLV-1 infected (both ATLL and AC subject groups); this observation could be explained by expansion of activated TEM (terminal effector memory) cells in ACs who have a relatively intact immune system, compared with the immune exhausted CTLs from ATLL patients. However, it is interesting to note that SAP levels were increased on HTLV-1 specific CD8+T cells compared to matched CD8+T cells in both HTLV-1 infected groups, yet an improvement in function was observed via CD107a degranulation and perforin expression on virus-specific CD8+T cells, thus, it is likely that SAP although increased could be dysfunctional or insufficient to mediate a stimulating response. It is also possible that the increased expression of 2B4 on these virus-specific CD8+T cells limits SAP function thus resulting in inhibition of effector function.

Thus, we demonstrate here, 2B4 up-regulation on HTLV-1 specific T cells, a downregulation of CD48 and an improvement in CD8+ T-cell function on ligand blockade.

Our study hereby supports an inhibitory role for the 2B4 receptor on HTLV-1 specific CD8+ T cells in HTLV-1 infection. The role of other co-inhibitory markers was not analyzed in the current study. Our findings show an association of 2B4 with functional impairment on virus specific CD8+ T cells in HTLV-1 infection. Immune therapies interfering with the 2B4-CD48 pathway with the aim of enhancing weak CTL responses and improving CTL function could be investigated as a novel treatment strategy for HTLV-1 infection.
